# ChAracterization of ItaliaN severe uncontrolled Asthmatic patieNts Key features when receiving Benralizumab in a real-life setting: the observational rEtrospective ANANKE study

**DOI:** 10.1186/s12931-022-01952-8

**Published:** 2022-02-19

**Authors:** Francesco Menzella, Elena Bargagli, Maria Aliani, Pietro Bracciale, Luisa Brussino, Maria Filomena Caiaffa, Cristiano Caruso, Stefano Centanni, Maria D’Amato, Stefano Del Giacco, Fausto De Michele, Fabiano Di Marco, Elide Anna Pastorello, Girolamo Pelaia, Paola Rogliani, Micaela Romagnoli, Pietro Schino, Gianenrico Senna, Alessandra Vultaggio, Lucia Simoni, Alessandra Ori, Silvia Boarino, Gianfranco Vitiello, Elena Altieri, Giorgio Walter Canonica

**Affiliations:** 1 Department of Medical Specialties, Pneumology Unit, Arcispedale Santa Maria Nuova, Azienda USL di Reggio Emilia-IRCCS, Reggio Emilia, Italy; 2 Respiratory Diseases and Lung Transplantation, Department of Medical and Surgical Sciences and Neurosciences, Siena University Hospital, Siena, Italy; 3 Respiratory Rehabilitation Unit of the Institute of Cassano Delle Murge, Istituti Clinici Scientifici Maugeri IRCCS, Bari, Italy; 4 Reparto di Pneumologia, Ospedale Ostuni, Ostuni, BR Italy; 5 Dipartimento di Scienze Mediche, SSDDU Allergologia e Immunologia Clinica, Università Degli Studi di Torino, AO Ordine Mauriziano Umberto I, Torino, Italy; 6 Cattedra e Scuola di Allergologia e Immunologia Clinica, Dipartimento di Scienze Mediche, Università di Foggia, Foggia, Italy; 7 Allergy Unit, Columbus Hospital, Fondazione Policlinico Universitario Agostino Gemelli IRCCS, Rome, Italy; 8 Respiratory Unit, Department of Health Sciences, ASST Santi Paolo e Carlo, Università Degli Studi di Milano, Milan, Italy; 9 UOSD Malattie Respiratorie “Federico II”, Ospedale Monaldi, AO Dei Colli, Napoli, Italy; 10 Department of Medical Sciences and Public Health, University of Cagliari, Cagliari, Italy; 11 UOC Pneumologia e Fisiopatologia Respiratoria, AORN A. Cardarelli, Napoli, Italy; 12 Department of Health Sciences, University of Milan, Pneumology, ASST Papa Giovanni XXIII, Bergamo, Italy; 13 Allergy and Immunology, Niguarda Hospital, Milano, Italy; 14 Dipartimento di Scienze Della Salute, Università Magna Graecia, Catanzaro, Italy; 15 Division of Respiratory Medicine, University Hospital “Tor Vergata”, Rome, Italy; 16 UOC Pneumologia, ULSS 2 Marca Trevigiana, Treviso, Italy; 17 Miulli Hospital, Acquaviva Delle Fonti, Bari, Italy; 18 Department of Medicine, University of Verona, Verona, Italy; 19 Immunoallergology Unit, Careggi University Hospital, Florence, Italy; 20 Medineos Observational Research, Viale Virgilio 54/U, 41123 Modena, Italy; 21 AstraZeneca Italia, Basiglio, MI Italy; 22 Reparto di Pneumologia, P.O. Garbagnate Milanese, Garbagnate Milanese, MI Italy; 23 Centro di Medicina Personalizzata: Asma e Allergia, Humanitas Clinical and Research Center, IRCCS, Rozzano, MI Italy; 24 Unit of Respiratory Medicine, Department of Experimental Medicine, University of Rome “Tor Vergata”, Rome, Italy; 25 Allergy Unit and Asthma Center, Verona University Hospital, Verona, Italy

**Keywords:** Benralizumab, Exacerbations, OCS, Real world, Severe eosinophilic asthma

## Abstract

**Background:**

Data from phase 3 trials have demonstrated the efficacy and safety of benralizumab in patients with severe eosinophilic asthma (SEA). We conducted a real-world study examining the baseline characteristics of a large SEA population treated with benralizumab in clinical practice and assessed therapy effectiveness.

**Methods:**

ANANKE is an Italian multi-center, retrospective cohort study including consecutive SEA patients who had started benralizumab therapy ≥ 3 months before enrolment (between December 2019 and July 2020), in a real-world setting. Data collection covered (1) key patient features at baseline, including blood eosinophil count (BEC), number and severity of exacerbations and oral corticosteroid (OCS) use; (2) clinical outcomes during benralizumab therapy. We also conducted two post-hoc analyses in patients grouped by body mass index and allergic status. Analyses were descriptive only.

**Results:**

Of 218 patients with SEA enrolled in 21 Centers, 205 were evaluable (mean age, 55.8 ± 13.3 years, 61.5% females). At treatment start, the median BEC was 580 cells/mm^3^ (interquartile range [IQR]: 400–850); all patients were on high-dose inhaled controller therapy and 25.9% were on chronic OCS (median dose: 10 mg/die prednisone-equivalent [IQR: 5–25]); 92.9% experienced ≥ 1 exacerbation within the past 12 months (annualized exacerbation rate [AER] 4.03) and 40.3% reported ≥ 1 severe exacerbation (AER 1.10). During treatment (median duration: 9.8 months [IQR 6.1–13.9]; ≥ 12 months for 34.2% of patients), complete eosinophil depletion was observed; exacerbation-free patients increased to 81% and only 24.3% reported ≥ 1 severe event. AER decreased markedly to 0.27 for exacerbations of any severity (− 93.3%) and to 0.06 for severe exacerbations (− 94.5%). OCS therapy was interrupted in 43.2% of cases and the dose reduced by 56% (median: 4.4 mg/die prednisone-equivalent [IQR: 0.0–10.0]). Lung function and asthma control also improved. The effectiveness of benralizumab was independent of allergic status and body mass index.

**Conclusions:**

We described the set of characteristics of a large cohort of patients with uncontrolled SEA receiving benralizumab in clinical practice, with a dramatic reduction in exacerbations and significant sparing of OCS. These findings support benralizumab as a key phenotype-specific therapeutic strategy that could help physicians in decision-making when prescribing biologics in patients with SEA.

*Trial registration* ClinicalTrials.gov Identifier: NCT04272463

**Supplementary Information:**

The online version contains supplementary material available at 10.1186/s12931-022-01952-8.

## Background

Severe asthma (SA) is defined by the inability to control symptoms despite treatment with high-dosage inhaled corticosteroids (ICS) plus a second controller and/or systemic corticosteroids [1]. SA affects up to 10% of the asthmatic population [1, 2] and, in approximately half [3], it presents with an eosinophilic phenotype (i.e., severe eosinophilic asthma, SEA) characterized by high blood and/or airway eosinophilia and persistent eosinophilic inflammation, recurrent exacerbations, lung function decline, and poor asthma control [4–6].

Albeit maintenance therapy with oral corticosteroids (OCS) has long been the mainstay to control symptoms and prevent exacerbations, their long-term use exposes patients to a significant dose-dependent risk of important sequelae, a higher risk of mortality and considerable costs [7–14].

The advent of monoclonal antibodies (mAbs) targeting the interleukin-5 (IL-5) pathway has provided a new phenotype-specific approach. Unlike the anti-IL-5 mAbs mepolizumab [15] and reslizumab [16], benralizumab [17] targets IL-5 receptor alpha (IL-5Rα), which is highly expressed by human eosinophils and eosinophil progenitors [6], and has the unique ability to induce rapid and complete depletion of blood and tissue eosinophils, and of other IL-5Rα + cells as well, via afucosylation-dependent Ab-dependent cell-mediated cytotoxicity (ADCC) [18, 19]. In the pivotal phase 3 trials, benralizumab as add-on maintenance treatment in patients with uncontrolled SEA decreased the annual exacerbation rate (AER) by up to 70%, improved lung function and asthma symptoms, and allowed a 75% reduction in the median dose of OCS (discontinuation rate: 52%), with a good safety and tolerability profile [20–22]. Notably, this benefit persisted for up to 5 years of continuous treatment [23–26].

Real-world evidence complements data from randomized controlled trials (RCTs) in a population fully representative of clinical practice [27]. However, the studies testing the effectiveness of benralizumab are small retrospective analyses and case reports [28–39], with the exception of two recent observational studies including 111 [39] and 130 patients [29], both reporting significant improvements in all outcome measures, including exacerbation rate and OCS consumption.

To expand the knowledge on the effectiveness of benralizumab in a large cohort of patients with SEA, we undertook the ANANKE study. Here, we describe the baseline characteristics of the patient population and the clinical outcomes observed after at least 3 months of treatment.

## Methods

### Study design

ANANKE (ClinicalTrials.gov Identifier: NCT04272463) is an Italian multi-center, observational retrospective cohort study including patients with SEA who had started benralizumab therapy as per clinical practice or within the Italian sampling program, activated after benralizumab approval in January 2018 and before reimbursement [17]. At the time of activation of the sampling program, clinical data on patients exposed to benralizumab were already available. This has permitted to collect data and meet the primary endpoint in a relatively short period of time after enrollment initiation.

Patients were enrolled consecutively during regular visits between December 2019 and July 2020 at 21 sites distributed across the country. The start date of benralizumab treatment was defined as the “index date”; the enrolment visit corresponded to the end of each patient’s retrospective observation window and occurred at least 3 months after the index date. Therefore, per protocol, data collection covered a period of > 15 months (i.e., 12 months before the index date) to retrieve a restricted set of clinical data plus at least 3 months between the index date and the enrolment visit (Additional file 1: Fig. S1).

ANANKE was performed in accordance with the principles of the Declaration of Helsinki and with the regulations and guidelines governing medical practice and ethics in Italy. Ethical approval was provided by the ethics committees/institutional review boards at each participating site (first approval: *n 248/19Sept2019—Università Magna Graecia, Catanzaro, Italy)*.

### Study population

Inclusion criteria were (1) age ≥ 18 years; (2) diagnosis of SEA requiring stable treatment with high doses of ICS and a long-acting β2-agonist (LABA) ± additional asthma controller; 3) start of benralizumab treatment at least 3 months before enrolment, either within the sampling program or as per routine practice, and at least one injection performed during this period (30 mg every 4 weeks for the first three doses, and then 30 mg every 8 weeks). Patients who temporarily or permanently interrupted benralizumab therapy before the enrolment visit may be included. Those who received benralizumab in a clinical experimental trial or participated in studies imposing a specific patient management strategy that did not correspond to the site’s normal clinical practice during the observation period were excluded.

All patients provided written informed consent before study entry.

### Outcomes and variables

After each patient had signed the informed consent and privacy form, data were collected from each hospital’s medical charts according to clinical practice and were entered into the electronic case report form (eCRF) by the study investigators.

#### Primary endpoint

The primary endpoint was to describe the patients’ key features recorded at the index date. As already stated, data collection started 12 months before the index date and included the following information, in addition to demographics, life habits and physical examination:

- *Comorbidities* such as rhinitis (allergic and non-allergic), gastroesophageal reflux, chronic obstructive pulmonary disease (COPD), sinusitis, chronic rhinosinusitis, nasal polyposis, atopic dermatitis, other eosinophilic conditions, conditions related to chronic OCS use (osteoporosis, cataract, etc.) *and other conditions deemed as relevant by the treating physician* at the index date;
- *Clinical assessments*: total IgE and blood eosinophil count (BEC) at the index date;
- *Number and severity of asthma exacerbations, defined according to clinical judgement,* that occurred in the 12 months before starting benralizumab. Severe exacerbations were defined as worsening of asthma that led to one of the following: i) use of systemic corticosteroids for ≥ 3 days or a temporary increase in a stable, background dosage of OCS; ii) an emergency department (ED) or urgent care visit (< 24 h) due to asthma that required systemic corticosteroids; or iii) admission to hospital (≥ 24 h) due to asthma. AER for any and severe exacerbations was calculated for these patients.
- *Previous asthma medications*, including *maintenance treatments* at the index date and *biologics* during the 12 months before the index date;
- *Lung function assessments*: forced expiratory volume in 1 s (FEV_1_), FEV_1_% of predicted, forced vital capacity (FVC), FEV_1_/FVC, fractional exhaled nitric oxide (FeNO)—both pre- and post-bronchodilator, if available, at the index date;
- *Patient-reported outcomes (PROs)*: asthma control test (ACT) questionnaire score [40, 41] and Asthma Quality of Life Questionnaire (AQLQ) score [42] at the index date;
- *Healthcare resource utilization,* in terms of general practitioner (GP)/specialist visits, ED admissions, and hospitalizations in the 12 months before the index date.

#### Secondary endpoint

The secondary endpoint relied on the description of the outcomes recorded during benralizumab treatment between the index date and the enrolment visit; when available, data at 16, 24 and 48 weeks after the index date were retrieved:

- *Patients’ adherence to benralizumab therapy* calculated as the ratio (as a percentage) between the number of actual injections received during the observation period and the number of expected injections, estimated based on the summary of product characteristics (SmPC)*, benralizumab discontinuation and the reasons leading to discontinuation and subsequent biologic treatments* during the observation period;
- *BEC and IgE levels and changes over time* with respect to the start of benralizumab treatment;
- Exacerbations of any severity, *severe exacerbations and their AER* during the observation period;
- *ICS and OCS reduction during the observation period;*
- *Lung function parameters and changes over time*;
- *PROs and changes over time;*
- *Healthcare resource utilization* (i.e., GP/specialist visits, ED admissions and hospitalizations) during the observation period.

### Post-hoc analyses

A post-hoc analysis of the SIROCCO and CALIMA studies showed that elevated BEC only, but not serum IgE levels, do increase the risk of exacerbations in patients with SEA [43]. We therefore conducted a post-hoc analysis to evaluate the performance of benralizumab according to the patients’ allergic status (i.e., positivity or not to the skin prick test for perennial allergens). Moreover, we performed a post-hoc analysis of obese vs overweight vs underweight/normal body mass index (BMI) patients. Indeed, a higher BMI is a risk factor for SA and, in particular, obesity is considered a chronic inflammatory disease with a negative effect on pulmonary ventilation and quality of life [44].

Outcome measures were OCS use, the median (interquartile range [IQR]) OCS dose reduction, and the AER of any/severe exacerbations before and after benralizumab treatment.

### Statistical analyses

Sample size was defined based on feasibility considerations: indeed, according to the volume of patients managed by the sites involved, inclusion of 200 participants (10 patients/site on average) meeting the inclusion/exclusion criteria was deemed as reasonable during the planned 8-month enrolment period. An evaluation of the possible achievable precision of the estimates for the primary analysis was performed considering the primary objective of the study and the literature data, when available. Assuming that 20% of enrolled patients could be non-eligible and/or non-evaluable for the primary analysis, the total number of evaluable patients would have been 160. With a sample size of 160 patients, an observed frequency of comorbidities and asthma treatments > 21% would have had an acceptable precision (i.e., relative error < 30%).

As all objectives were descriptive in nature, analyses were descriptive only, and were carried out using the mean, standard deviation (SD), median, IQR, range, and absolute and relative frequencies. No statistical method was applied to check for normality of data distribution. The Median and IQR were preferred over mean (SD) in case of high variability in data distribution (i.e., high SD or high difference between mean and median). Per protocol, evaluable patients with missing data for certain variables were not excluded from the study and their data were not replaced, although they were not considered for the analyses including those variables. The frequency of missing data was given for all the variables analyzed. No formal hypotheses were prespecified. The analyses were performed using SAS software (SAS Institute, Cary, NC, USA).

## Results

### Patient disposition

Between December 2019 and July 2020, 218 patients were enrolled in 21 centers across all regions in the country: 211 were fully eligible whereas 7 were excluded because benralizumab was started < 3 months before enrolment (N = 2), no LABA treatments (either in the 12 months before starting benralizumab, or during the observation period; N = 1), or incomplete/inconsistent data entry in the eCRF for relevant fields (N = 4). Six more patients were excluded because of the lack of information regarding BEC at the start of benralizumab treatment. Thus, the complete evaluable set comprised 205 (94.0%) patients with SEA, which fulfils the required sample size.

### Baseline characteristics

Patient demographics and clinical characteristics recorded at the index date or during the previous 12 months are presented in Tables 1 and 2.

**Table 1:** Patient characteristics recorded before the start of benralizumab therapy (i.e., at the index date or during the 12 months prior to the index date)

Characteristics	Evaluable population N = 205
Age at the index date, yrs	55.8 ± 13.3
Female sex	126 (61.5%)
BMI at the index date, kg/m^2^ (N = 182)	
Under/Normal weight	70 (38.5%)
Overweight	79 (43.4%)
Obese	33 (18.1%)
Smoking status at the index date (N = 195)	
Non-smoker	139 (71.3%)
Previous smoker	50 (25.6%)
Current smoker	6 (3.1%)
Age at asthma diagnosis, yrs (N = 203)	38.9 ± 16.7
Asthma duration at the index date, yrs (N = 203)	12.4 (6.3–24.6)
SEA duration at the index date, yrs (N = 203)	1.6 (1.0–3.1)
Atopy at the index date	85 (41.5%)
Comorbidities at the index date	
≥ 1 current asthma-related condition	103 (50.2%)
Chronic rhinosinusitis	50 (24.4%)
GERD	43 (21%)
Allergic conjunctivitis	28 (13.7%)
Allergic rhinitis	45 (22%)
Other (atopic dermatitis, urticaria, etc.)	17 (8.3%)
≥ 1 current OCS-related condition	77 (37.6%)
Hypertension	46 (22.4%)
Osteoporosis	23 (11.2%)
Cataract	12 (5.9%)
Anxiety/Depression	11 (5.3%)
Type 2 Diabetes Mellitus	10 (4.9%)
Obstructive sleep apnoea	10 (4.9%)
Cardiovascular disease	7 (3.4%)
Other OCS-related ongoing comorbidities	19 (9.3%)
≥ 1 other ongoing comorbidities	35 (17.1%)
Thyroid disorders	8 (3.9%)
Bronchiectasis	6 (2.9%)
Blood eosinophil count at the index date, cells/mm^3^	580 (400–850)
Total serum IgE at the index date, IU/mL (N = 123)	289 (85–573)
Exacerbations during the 12 months prior to the index date (N = 196)	
≥ 1, any severity	182 (92.9%)
AER	4.03
≥ 1 mild	101 (51.5%)
≥ 1 moderate	121 (61.7%)
≥ 1 severe	79 (40.3%)
AER	1.10

Data are N (%), mean ± SD, or median (IQR). Unless otherwise stated, the evaluable population included 205 patients

*yrs* years, *BMI* body mass index, *SEA* severe eosinophilic asthma, *GERD* gastroesophageal reflux disease, *OCS* oral corticosteroids, *AER* annual exacerbation rate

**Table 2:** Data on prior asthma medication, lung function, patient-recorded outcomes and healthcare utilization recorded before the start of benralizumab therapy (i.e., at the index date or during the 12 months prior to the index date)

Characteristics	Evaluable population N = 205
Corticosteroid use at the index date	
ICS/LABA or ICS + LABA	
Any ICS dose	205 (100%)
Low ICS dose	3 (1.5%)
Low/Medium ICS dose	16 (7.8%)
Medium ICS dose	6 (2.9%)
High ICS dose	179 (87.3%)
Data unknown	1 (0.5%)
LAMA	104 (50.7%)
Other*	88 (42.9%)
OCS	53 (25.9%)
OCS dosage among users, prednisone-equivalent, mg/die (N = 48)	10 (5–25)
Biologic use during the 12 months prior to the index date	
Yes, any biologic	58 (28.3%)
Omalizumab	34 (16.6%)
Mepolizumab	19 (9.3%)
Omalizumab > Mepolizumab	5 (2.4%)
No	147 (71.7%)
Lung function at the index date	
Pre-bronchodilator FEV_1_, L (N = 154)	2.0 ± 0.8
Post-bronchodilator FEV_1_, L (N = 92)	2.1 ± 0.9
Pre-bronchodilator FEV_1_, % predicted (N = 159)	70.6 ± 21.6
Post-bronchodilator FEV_1_, % predicted (N = 90)	75.3 ± 22.9
Pre-bronchodilator FVC, L (N = 148)	3.0 ± 1.0
FeNO, ppb (N = 66)	46.7 ± 34.6
ACT score at the index date (N = 161)	14.7 ± 4.7
AQLQ score at the index date (N = 32)	3.7 ± 1.2
Healthcare resource utilization for asthma per patient during the 12 months prior to the index date (N = 189)	
Primary care physician/GP office visits	1.0 ± 2.0
Specialist visits	2.4 ± 3.0
ED admissions	0.2 ± 0.5
Hospitalizations	0.1 ± 0.4

Data are N (%), mean ± SD, or median (IQR). Unless otherwise stated, the evaluable population included 205 patients

*ICS* inhaled corticosteroid, *LABA* long-acting β2-agonist, *LAMA* long-acting muscarinic receptor antagonist, *FEV*_*1*_ forced expiratory volume in 1 s, *FVC* forced vital capacity, *FeNO* fractioned exhaled nitric oxide ACT, asthma control test, *AQLQ* asthma quality of life questionnaire, *GP* general practitioner, *ED* emergency department

Patients (mean age, 55.8 ± 13.3 years, 126 [61.5%] females) were mostly overweight/obese (112/182, 61.5%) and non-smokers (139/195, 71.3%); 101 (49.3%) subjects were positive for ≥ 1 seasonal/perennial allergen and 85 (41.5%) for ≥ 1 perennial allergens, and, in particular, 63 (30.7%) to house dust mite (*D. Pteronyssinus*), 20 (9.8%) to cat dander, 16 (7.8%) to dog dander, 16 (7.8%) to *Aspergillus*, and 5 (2.4%) to mould mix. The median duration of asthma and SEA at the index date was 12.4 years (IQR 6.3–24.6) and 1.6 (IQR 1.0–3.1), respectively.

Of 175 (85.4%) patients with relevant comorbidities, 110 (53.7%) had current or past positive history for nasal polyposis, 103 (50.2%) had current asthma-related conditions (chronic rhinosinusitis in 50 [24.4%], allergic rhinitis in 45 [22.0%], and gastroesophageal reflux in 43 [21.0%]), and 77 (37.6%) had OCS-related conditions (hypertension in 46 [22.4%] and osteoporosis in 23 [11.2%]).

**Fig. 1:**
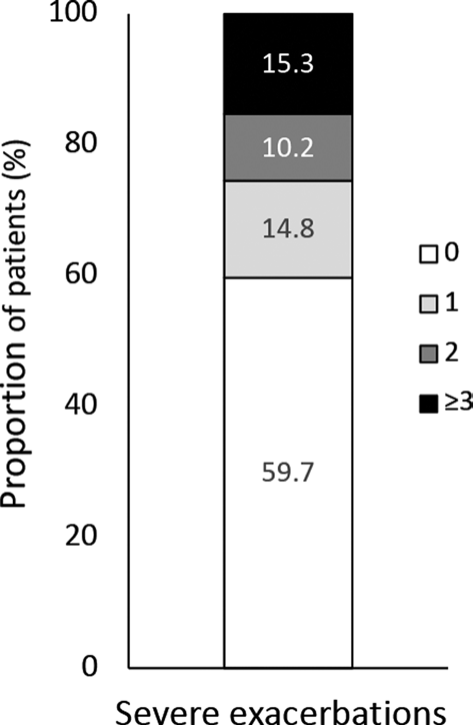
Distribution of patients according to the number of severe exacerbations experienced in the 12 months prior to the index date. Evaluable patients with information on previous exacerbations were 196

Before the start of benralizumab treatment, the median BEC was 580 cells/mm3 (IQR 400–850) and the median level of IgE was 289 IU/mL (IQR 85–573). All patients were on maintenance treatment with ICS/LABA or ICS + LABA (the dose of ICS was high in most cases), and 53 (25.9%) reported chronic OCS use, with a median dose of 10 mg/die prednisone-equivalent (IQR: 5–25). Nonetheless, lung function was suboptimal and asthma control and quality of life were poor, and of 196 patients with available data, only 14 (7.1%) were exacerbation-free at the index date,^1^ whereas 182 (92.9%) had experienced ≥ 1 exacerbation of any severity with an AER of 4.03, and 79 (40.3%) had experienced ≥ 1 severe exacerbation with an AER of 1.10 (Table 1 and Fig. 1).

Last, in the 12 months prior to the index date, 53 (25.9%) patients had received one biologic (median time gap between previous biologic end and benralizumab start: 2.9 months [IQR 1.4–9.5]), which was omalizumab in most cases, and 5 (2.4%) had received two different biologics, omalizumab followed by mepolizumab; however, because of uncontrolled disease, these patients were switched to benralizumab. A post-hoc analysis will be provided on this subgroup of patients.

### Parameters recorded during benralizumab treatment

#### Benralizumab exposure, adherence, and persistence on treatment

Among 202 evaluable patients, 198 (98.0%) were still on treatment with benralizumab at enrolment; the median (IQR) duration of exposure was 9.8 months (IQR 6.1–13.9); 69 (34.2%) patients received therapy for at least 12 months.

The median number of actual injections administered per patient was 7.0 (IQR 5.0–9.0); deviations from the SmPC were recorded for 24/190 (12.6%) evaluable patients; the median level of patients’ adherence was 100% (range 66.7–100%).

Only 4 patients discontinued therapy permanently (after a median of 5 months [IQR 1.4–11.9], due to patient decision (N = 2, 1.0%), lack of clinical efficacy (N = 1, 0.5%) or physician decision (N = 1, 0.5%). Because this study is retrospective, reasons for patient or physician decisions for discontinuation were not collected. Additional file 2: Fig. S2 shows persistence on benralizumab treatment.

After discontinuing benralizumab, 1 patient switched to another biologic (omalizumab) because of inadequate clinical response in terms of exacerbations, FEV_1_ deterioration and symptom recurrence.

Finally, of 194 patients evaluable with the dose of ICS available at the index date, 179 (92.3%) did not reduce the dose of ICS during treatment with benralizumab, while 15 (7.7%) did (any reduction of any extent).

#### Eosinophil count and IgE level

**Fig. 2:**
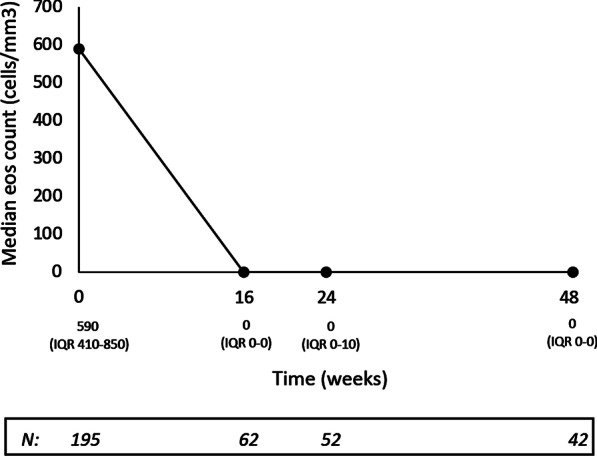
Median blood eosinophil count (cells/mm^3^) recorded at the index date (week 0) and during benralizumab treatment, up to week 48

During benralizumab treatment, compared with the index date, the BEC fell to 0 (IQR 0.0–0.0) at week 16 and remained low thereafter (Fig. 2). As for the level of IgE, the limited number of patients with data available at the prespecified time points (N = 9 at 16 weeks; N = 12 at 24 weeks; N = 4 at 48 weeks; N = 1 at enrolment) did not allow the extent of variation over time to be evaluated with sufficient precision.

#### Exacerbations

**Fig. 3:**
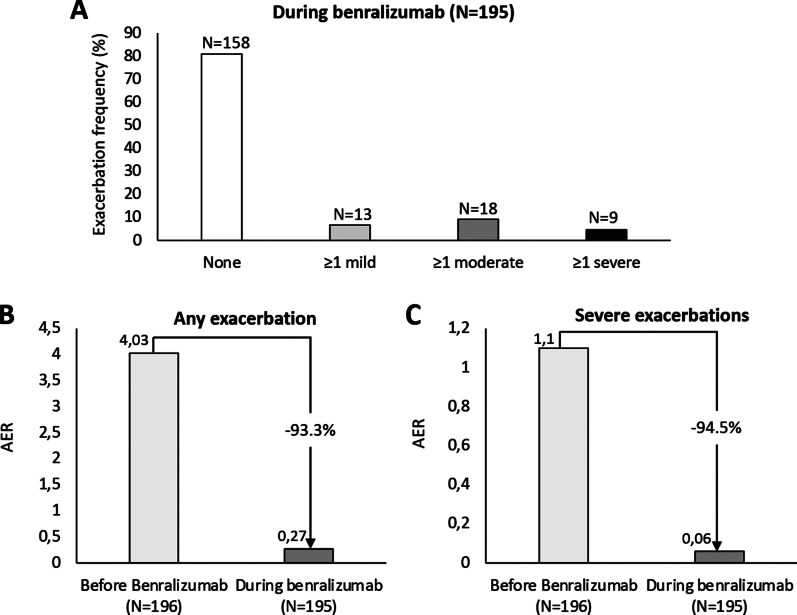
Data on exacerbations during benralizumab treatment. **A** Exacerbation occurrence. **B** Any exacerbation annual exacerbation rate (AER) variations **C** Severe exacerbation AER variations. Week 0 corresponds to the index date

During benralizumab treatment, as much as 158/195 (81%) patients reported zero exacerbations; of the 37 who had at least 1 exacerbation, only 9 (24.3%) experienced a severe event (8 patients reported 1 severe exacerbation and 1 reported 2 severe exacerbations) (Fig. 3A): 2 occurred between the index date and week 2, 5 by week 16, 1 at week 24, and 2 at week 48. All severe exacerbations resolved and benralizumab therapy was continued thereafter.

Moreover, compared with the index date, a marked decrease in AER was recorded for both any and severe exacerbations: from 4.03 to 0.27 for any, − 93.3%; from 1.1 to 0.06 for severe exacerbations, − 94.5% (Fig. 3B).

#### OCS use

**Fig. 4:**
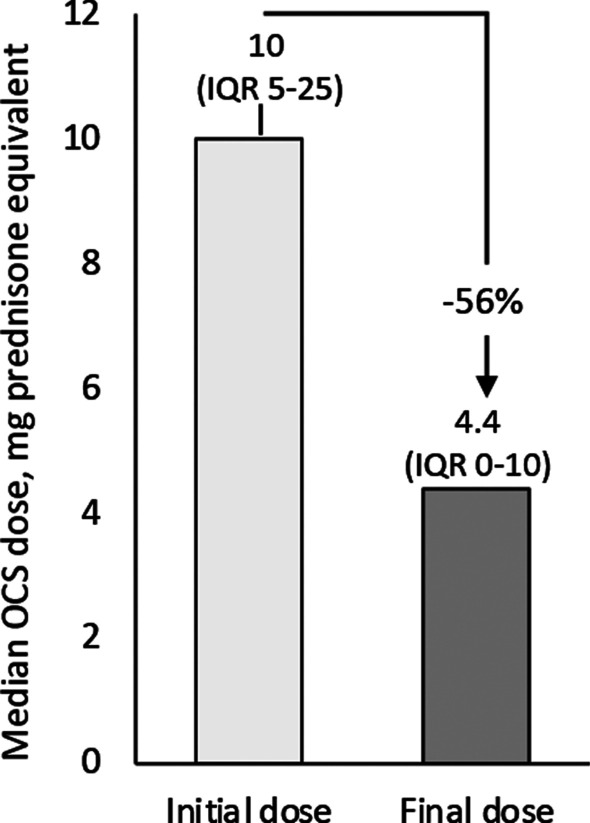
OCS dosage reduction during benralizumab treatment. Data are expressed as median in mg prednisone equivalent

Of 53 OCS users at baseline, 9 patients maintained their dose and 44 had data documenting dose variations during benralizumab treatment. Therapy with OCS was reduced in as much as 22 of 44 patients (50%) and interrupted in 19 (43.2%), with similar rates between patients who had started on a dose ≤ 5 mg/die vs > 5 mg/die prednisone-equivalent (Table 3). The overall dose reduction was of 56%, from a median OCS dose of 10.0 mg/die prednisone-equivalent (IQR 5.0–25.0) at benralizumab start to 4.4 mg/die prednisone-equivalent (IQR 0.0–10.0) during treatment (Fig. 4). Dose reduction was independent of eosinophil count at the index date, age at asthma onset, IgE level and number of exacerbations recorded in the previous 12 months (data not shown).

**Table 3:** Changes in OCS use (dosage expressed as prednisone-equivalent) during treatment with benralizumab

Variable	Evaluable N = 44	Starting dose ≤ 5 mg/die (N = 14)	Starting dose > 5 mg/die (N = 30)
Any reduction	22 (50%)	8 (57.1%)	14 (46.7%)
Reduction from baseline			
100%	19 (43.2%)	6 (42.9%)	13 (43.3%)
≥ 90%	19 (43.2%)	6 (42.9%)	13 (43.3%)
≥ 75%	20 (45.5%)	6 (42.9%)	14 (46.7%)
≥ 25%	21 (47.7%)	7 (50%)	14 (46.7%)
No reduction	22 (50%)	6 (42.9%)	16 (53.3%)

Data are expressed as frequencies (N [%])

#### Lung function and asthma control

**Fig. 5:**
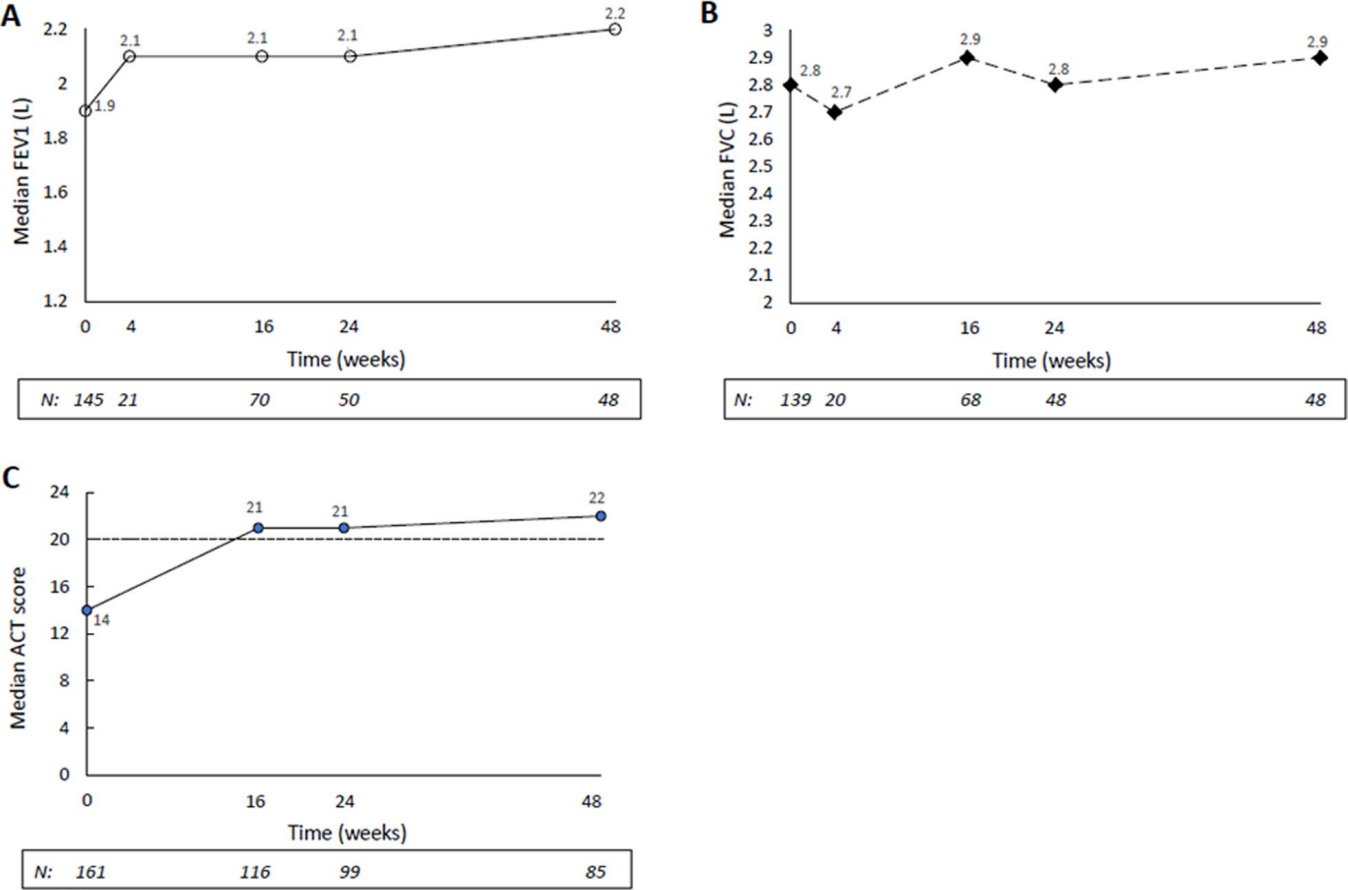
Outcome measures recorded at the index date (week 0) and during benralizumab treatment, up to week 48. **A** FEV_1_ (L); **B** FVC (L); **C** ACT score

During treatment with benralizumab, we observed an improvement in lung function (at week 48 vs the index date: median FEV_1_, + 300 mL and median FVC, + 100 mL; Fig. 5A, B), and in asthma control (median ACT: from 14 at the index date to 22 at week 48; Fig. 5C). In particular, for patients with available data, the proportion of patients with well-controlled asthma (i.e., ACT score ≥ 20) increased over time from 16.8% (N = 27) at week 0 (index date) to 65.5% (N = 76) at week 16, 70.7% (N = 70) at week 24 and 81.2% (N = 69) at week 48, and, at every evaluation during benralizumab treatment, ≥ 75% of the patients with completed questionnaires achieved the minimum important difference from baseline. No evaluation could be made for the AQLQ score, due to incomplete questionnaires as filled out by patients in actual clinical practice.

#### Health care resource utilization

During the observation period (mean: 10.3 ± 5 months), 190 (97.4%) patients had no ED admissions and 191 (97.9%) had no hospitalizations. Variations recorded during benralizumab therapy are reported in Table 4.

**Table 4:** Variations of healthcare resource utilization recorded during benralizumab therapy

Item	After benralizumab
Primary care physician/GP office visits for asthma per patient	0.42 − 58%
Specialist visits for asthma per patient	2.35 − 2.1%
ED admissions for asthma per patient	0.025 − 87.5%
Hospitalizations for asthma per patient	0.021 − 79%

Data are expressed as mean and % of variation vs index date. Evaluable patients, N = 195

*GP* general practitioner, *ED* emergency department

### Post-hoc analyses

#### Allergic vs non-allergic patients

Allergic patients and non-allergic were 85/205 (41.5%) and 120/205 (58.5%), respectively. The main characteristics recorded at the index date and in the 12 months before are reported in Additional file 5: Table S1. The total duration of benralizumab exposure was 10.3 ± 4.5 months for allergic patients and 10.3 ± 5.3 months for non-allergic patients.

Data on the use of OCS are summarized in Additional file 6: Table S2. Among the evaluable allergic patients (N = 14), the rate of OCS reduction during benralizumab treatment was (42.9%, N = 6), and the rateof discontinuation was 35.7% (N = 5), with a 56% OCS dosage reduction. In evaluable non-allergic patients (N = 30), the corresponding rates were 53.3% (N = 16) and 46.7% (N = 14), with a 77% OCS dosage reduction.

In both groups, a remarkable decline in the AER of any and of severe exacerbations was observed: for any exacerbation, by − 91.8% in allergic patients and − 94.3% in non-allergic patients; for severe exacerbations, by − 91.9% and − 97%, respectively (Additional file 3: Fig. S3).

#### Obese vs overweight vs underweight/normal BMI patients

The main features recorded at the index date and in the 12 months before are reported in Additional file 7: Table S3. The total duration of benralizumab exposure was 10.4 ± 4.8 months for obese patients, 10.1 ± 4.6 months for overweight patients and 10.2 ± 5.4 months for underweight/normal BMI patients.

During benralizumab treatment, the AER of any exacerbation and of severe exacerbations decreased considerably in all groups: for any exacerbation, by − 90.9% in obese patients, − 92.0% in overweight and − 96.0% in underweight/normal BMI patients; for severe exacerbations, by − 92.9%, − 91.1% and − 97.0%, respectively (Additional file 4: Fig. S4). Data on OCS use in these subgroups of patients were too small to be reported and analyzed.

## Discussion

ANANKE describes the baseline characteristics of a large cohort of 205 patients with uncontrolled SEA receiving benralizumab in clinical practice. Moreover, it further supports the effectiveness of benralizumab in terms of exacerbation reduction, OCS sparing, lung function improvement and asthma control. To our knowledge, this is the largest cohort published so far.

Five biologics targeting the IL-5, IgE and IL-4/IL-13 pathways are currently available for the treatment of patients with SEA. No direct comparison exists and the differences in the trial populations hamper any indirect comparisons. According to the treatment algorithm provided in the 2020 European Academy of Allergy and Clinical Immunology biologicals guidelines, the decision relies on the combination of phenotypic traits, biomarkers and clinically relevant asthma-related end points [45]. A strong recommendation on the use of all biologicals has been issued with regard to the decrease in SA exacerbations, and on the use of benralizumab (if BEC > 150/μL) and mepolizumab for decreased use of withdrawal of OCS.

Recent analyses have identified BEC ≥ 300 cells/μL, the presence of nasal polyposis, adult-onset asthma, OCS use, compromised lung function, and a history of ≥ 3 exacerbations/year at baseline as predictors of enhanced response to benralizumab, particularly for reduction in exacerbations and improvement in lung function [43, 46–48]. The phenotype of the ANANKE population (median BEC: 589 [IQR 400–850]; nasal polyposis: 53.7%; mean age at asthma diagnosis: 38.9 ± 16.7 years; OCS use: 25.9%; FEV_1_: 70.6% ± 21.6% of predicted; ≥ 3 exacerbations/year: 15.3%) likely accounts for the observed effectiveness of benralizumab.

The unique mechanism of action of this mAb (i.e., anti-IL-5Rα and ADCC) explains the rapid (as early as 24 h after the start of treatment) [37] and complete depletion of eosinophils observed in treated patients with SEA. Eosinophils play a central role in asthma pathogenesis by inducing structural remodeling of small airways and airflow limitations; moreover, high eosinophil counts are important predictors of the risk of exacerbations [43]. In keeping with these data, compared with baseline, we observed complete eosinophil suppression, which was sustained throughout week 48, together with an impressive drop of > 93% in the AER of any and severe exacerbations, and a remarkable increase in the proportion of any exacerbation-free patients, from 7.1 to 81%. Importantly, severe exacerbations decreased from 79 to 9 events, and those reported during treatment resolved without interruption of therapy. In the observational study by Pelaia et al. [39], a significant reduction in the rate of exacerbations was observed after 24 weeks of treatment, from 4 (3–6) to 0 (0–0). Moreover, Kavanagh et al. documented a 72.8% reduction in AER (from 4.92 ± 3.35 to 1.34 ± 1.71, P < 0.001) after 48 weeks of benralizumab therapy [29].

Albeit the use of OCS remains a matter of concern, a considerable proportion of patients with SA still rely on OCS therapy, often at high doses [49, 50]. In the present study, 25% of SEA patients used OCS regularly (and 37.6% had OCS-related conditions at baseline), a proportion lower than that reported by the Severe Asthma Network in Italy (SANI, N = 437) registry (62% to 64.1%) [7, 8] and by other real-world experiences [29, 32, 50] but similar to the recently published Italian registry on Severe Asthma (IRSA) [38, 51]. These discrepancies may depend on to the different definitions of OCS usage, e.g., OCS burst vs maintenance use vs chronic use. Regardless, in ANANKE, as much as 43.2% of patients discontinued OCS therapy and 50% reduced the dosage, with an overall dose reduction from baseline of 56%. These findings further support the OCS-sparing effect of benralizumab observed even in real-world settings [21, 25, 29, 39]. Indeed, Pelaia et al. [39] demonstrated a significant reduction in the dose, from 5 mg (IQR 0–12.5) to 0 mg (IQR 0–0), and in the proportion of patients on OCS, from 72 to 20%; Kavanagh et al. [29] documented a 100% reduction in the median dose of OCS, with a discontinuation rate of 51.4%.

During benralizumab treatment, we observed amelioration of lung function and asthma control, in line with previous studies. In ANANKE, the median FEV_1_ increased already at week 4, reaching a + 300 mL change at week 48. Pelaia et al. [39] observed a progressive, significant increase in FEV_1_ from month 1 (+ 390 mL) to month 6 (+ 530 mL), and Kavanagh et al. [29] reported a change in FEV_1_ of + 140 mL at 48 weeks.

All patients received benralizumab for at least 3 months and 34.2% for at least 12 months; treatment adherence was very high (likely favored by the ease of administration), with only 2% of patients permanently discontinuing therapy; this is important to ensure the rapid and sustained effects of the drug [29, 32, 36, 46].

Finally, the post-hoc analyses testing the performance of benralizumab according to the patients’ allergic status (allergic vs non-allergic) and BMI (obese vs overweight vs underweight/normal), demonstrated a benefit on both exacerbations (> 91% reduction in AER of any and severe exacerbations) and OCS use regardless of the presence of allergy, in line with previous reports [28, 32, 39, 52]. As for BMI, at the end of observation, a consistent AER reduction of > 90% was observed for both any and severe exacerbations, in all subgroups analyzed. These trends deserve further investigations in larger cohorts.

The main limitations of this study are linked to the retrospective, real-world study design. However, in our opinion, this also represents a strength, because the large sample size, the high number of centers involved throughout Italy and the comprehensive set of data collected make these results generalizable to a population of patients with SEA. We acknowledge that no sensitivity analyses have been performed to help identifying selection and center bias for the results. However, we think that the risk of inhomogeneity is minimized by the fact that benralizumab was prescribed according to the therapeutic plan issued by the Italian Medicines Agency in all the centers involved in this study.

Real-world evidence (RWE) studies are of increasing importance to both regulatory agencies and reimbursement authorities [53]. For these reasons too, our extensive real-life study can provide important and solid information not only for clinicians but also for decision-makers.

## Conclusion

In clinical practice, defining the patient profile is important to maximize treatment efficacy. Here, we describe in a large cohort of patients with uncontrolled SEA the set of characteristics identifying the patients treated with benralizumab in clinical practice and confirm that these patients attain a remarkable response in terms of exacerbations, OCS use, lung function and asthma control. Altogether, these findings contribute to make benralizumab a key phenotype-specific strategy and should help physicians in decision-making.

ANANKE will end in 2022 and, as part of a large program in other European countries, its results will contribute to a pan-European and Canadian 2-year evaluation of benralizumab treatment in clinical practice.

## Supplementary Information

**Additional file 1: Fig. S1**. Study design.

**Additional file 2: Fig. S2**. Persistence on benralizumab treatment: Kaplan-Meier survival analysis (eligible patientswith consistent data). Time from index date to treatment discontinuation (weeks) is the time elapsed between index date and date of benralizumab discontinuation (in case of patients permanently discontinuing treatment) or date of enrolment visit (in case of patients not permanently discontinuing treatment). The event is defined as the permanent discontinuation of the treatment. The patients who didn’t discontinue treatment during observation period were censored at date of enrolment visit.

**Additional file 3: Fig. S3**. Variations in the annualized exacerbation rate (AER) of (A) any exacerbation and (B) of severe exacerbations during benralizumab treatment, in allergic vs non-allergic patients.

**Additional file 4: Fig. S4**. Variations in the annualized exacerbation rate (AER) of (A) any exacerbation and of (B) severe exacerbations during benralizumab treatment, in obese vs overweight vs underweight/normal BMI patients.

**Additional file 5: Table S1**. Patient characteristics recorded before the start of benralizumab therapy. Data are N (%), mean±SD, or median (IQR). Unless otherwise specified, the evaluable populations included 85 allergic and 120 non-allergic patients.

**Additional file 6: Table S2**. Evaluable patients with data on OCS use at the index date and at enrolment are 14 for allergic and 30 for non-allergic subjects. OCS dose is indicated as a median (IQR).

**Additional file 7: Table S3**. Patient characteristics recorded before the start of benralizumab therapy. Data are N (%), mean±SD, or median (IQR). Unless otherwise specified, the evaluable populations included 33 obese, 79 overweight and 70 underweight/normal BMI patients.

## Data Availability

The datasets used and/or analyzed during the current study are available from the corresponding author on reasonable request.

## Abbreviations

ACT: Asthma control test
ADCC: Afucosylation-dependent Ab-dependent cell-mediated cytotoxicity
AER: Annual exacerbation rate
AQLQ: Asthma Quality of Life Questionnaire
BEC: Blood eosinophil count
BMI: Body mass index
COPD: Chronic obstructive pulmonary disease
eCRF: Electronic case report form
FDA: Food and Drug Administration
FeNO: Fractional exhaled nitric oxide
FEV_1_: Forced expiratory volume in 1 s
FVC: Forced vital capacity
GP: General practitioner
ICS: Inhaled corticosteroids
IL-5: Interleukin-5
IL-5Rα: IL-5 receptor alpha
IQR: Interquartile range
IRSA: Italian registry on Severe Asthma
LABA: Long-acting β2-agonist
mAbs: Monoclonal antibodies
OCS: Oral corticosteroids
PROs: Patient reported outcomes
RCTs: Randomized controlled trials
RWE: Real-world evidence
SA: Severe asthma
SANI: Severe Asthma Network in Italy
SD: Standard deviation
SEA: Severe eosinophilic asthma

## Footnotes

1 Benralizumab is reimbursed by the Italian healthcare system in adult patients with refractory SEA in the following situations: i) if they had ≥ 300 BEC in the absence of systemic steroid treatment; ii) if, despite maximum ICS therapy, they experienced in the previous 12 months ≥ 2 exacerbations treated with systemic steroids or requiring hospitalization OR if, in the previous 12 months, they received chronic systemic steroids, on top of the maximum ICS therapy.
