# Supplementary figures and images for: ChAracterization of ItaliaN severe uncontrolled Asthmatic patieNts Key features when receiving Benralizumab in a real-life setting: the observational rEtrospective ANANKE study
Source: Respir Res. 2022 Feb 19;23:36. doi: 10.1186/s12931-022-01952-8 (PMC8858449; doi:10.1186/s12931-022-01952-8)

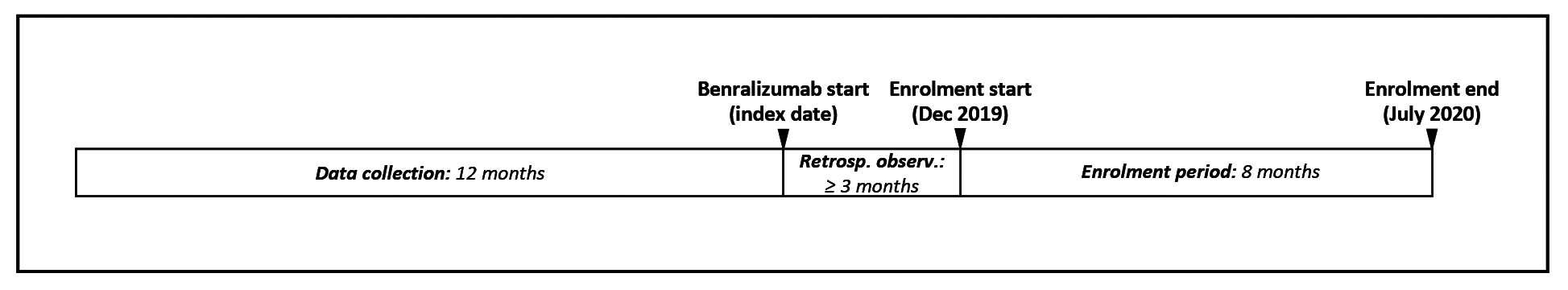

Supplement: Supplementary file 1 — Additional file 1: Fig. S1. Study design. [file 12931_2022_1952_MOESM1_ESM.tif]

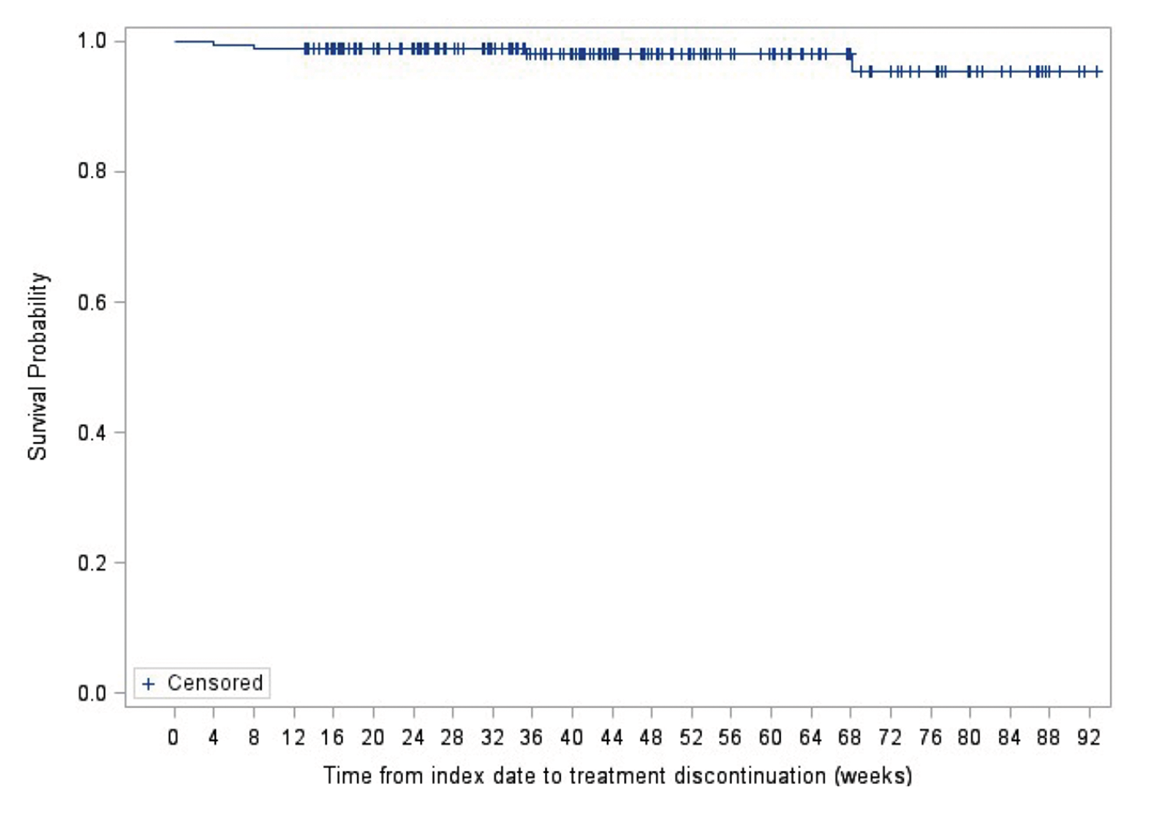

Supplement: Supplementary file 2 — Additional file 2: Fig. S2. Persistence on benralizumab treatment: Kaplan-Meier survival analysis (eligible patientswith consistent data). Time from index date to treatment discontinuation (weeks) is the time elapsed between index date and date of benralizumab discontinuation (in case of patients permanently discontinuing treatment) or date of enrolment visit (in case of patients not permanently discontinuing treatment). The event is defined as the permanent discontinuation of the treatment. The patients who didn’t discontinue treatment during observation period were censored at date of enrolment visit. [file 12931_2022_1952_MOESM2_ESM.tif]

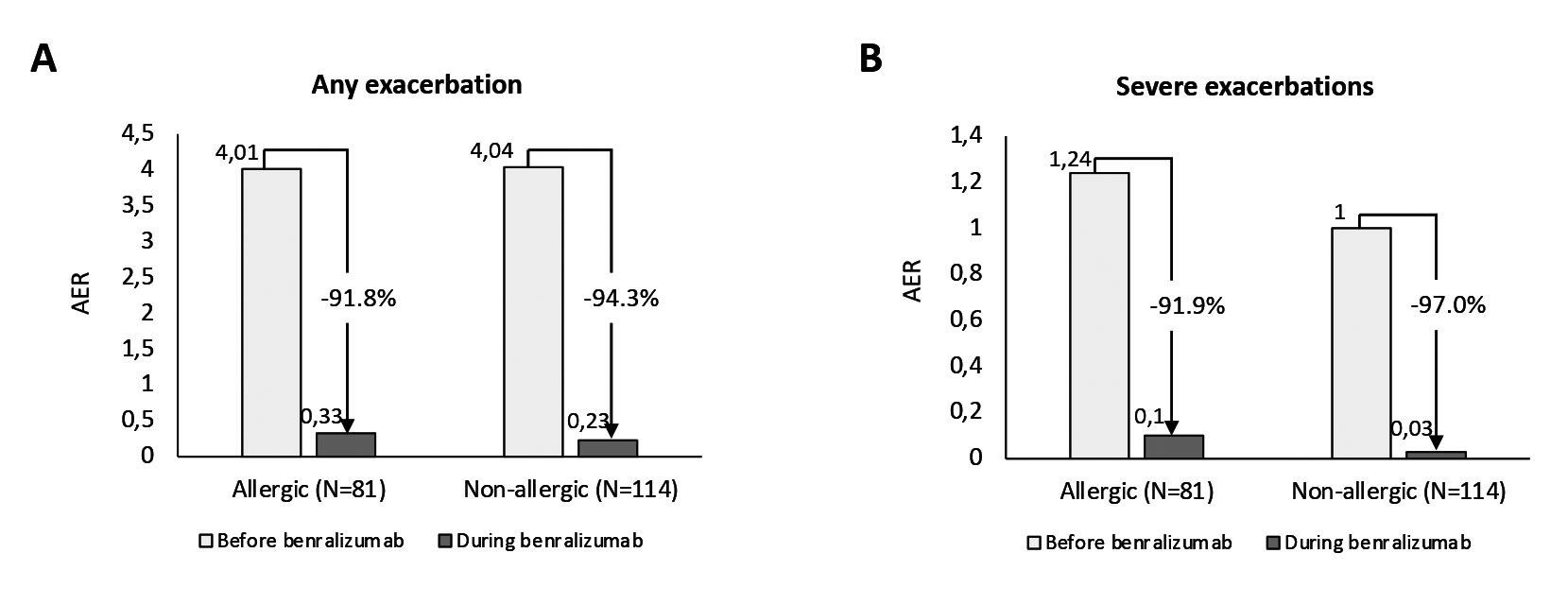

Supplement: Supplementary file 3 — Additional file 3: Fig. S3. Variations in the annualized exacerbation rate (AER) of (A) any exacerbation and (B) of severe exacerbations during benralizumab treatment, in allergic vs non-allergic patients. [file 12931_2022_1952_MOESM3_ESM.tif]

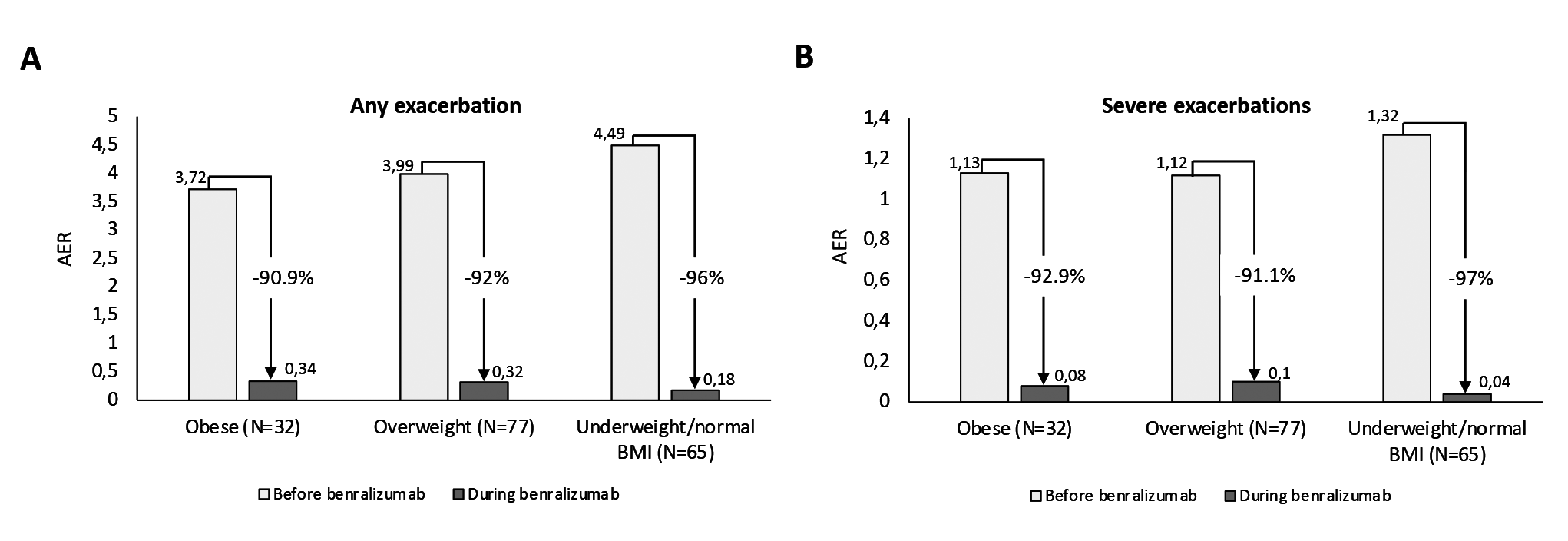

Supplement: Supplementary file 4 — Additional file 4: Fig. S4. Variations in the annualized exacerbation rate (AER) of (A) any exacerbation and of (B) severe exacerbations during benralizumab treatment, in obese vs overweight vs underweight/normal BMI patients. [file 12931_2022_1952_MOESM4_ESM.tif]
