# Supplementary material for: ChAracterization of ItaliaN severe uncontrolled Asthmatic patieNts Key features when receiving Benralizumab in a real-life setting: the observational rEtrospective ANANKE study
Source: Respir Res. 2022 Feb 19;23:36. doi: 10.1186/s12931-022-01952-8 (PMC8858449; doi:10.1186/s12931-022-01952-8)
Supplement: Supplementary file 5 — Additional file 5: Table S1. Patient characteristics recorded before the start of benralizumab therapy. Data are N (%), mean±SD, or median (IQR). Unless otherwise specified, the evaluable populations included 85 allergic and 120 non-allergic patients. [file 12931_2022_1952_MOESM5_ESM.docx]

**Additional Table 1.** Patient characteristics recorded before the start of benralizumab therapy. Data are N (%), mean±SD, or median (IQR). Unless otherwise specified, the evaluable populations included 85 allergic and 120 non-allergic patients.

| **Characteristics** | **Evaluable allergic**  **N=85** | **Evaluable non-allergic**  **N=120** |
| --- | --- | --- |
| **Age at the index date, yrs** | 52.6±13.5 | 58.1±12.7 |
| **Female sex** | 49 (57.6%) | 77 (64.2%) |
| **BMI at the index date, kg/m2 (N=74; N=108)** |  |  |
| Underweight/Normal weight | 27 (36.5%) | 43 (39.8%) |
| Overweight | 31 (41.9%) | 48 (44.4%) |
| Obese | 16 (21.6%) | 17 (15.7%) |
| **Smoking status at the index date (N=82; N=113)** |  |  |
| Non-smoker | 55 (67.1%) | 84 (74.3%) |
| Previous smoker | 22 (26.8%) | 28 (24.8%) |
| Current smoker | 5 (6.1%) | 1 (0.9%) |
| **Age at asthma diagnosis, yrs (N=84; N=119)** | 35.1±16.5 | 41.6±16.4 |
| **Asthma duration at the index date, yrs (N=84; N=119)** | 14.8 (6.3-27.4) | 11.0 (6.3-24.0) |
| **Comorbidities at the index date** | 74 (87.1%) | 101 (84.2%) |
| ≥1 current asthma-related condition | 52 (70.3%) | 51 (50.5%) |
| Chronic rhinosinusitis | 24 (32.4%) | 26 (25.7%) |
| GERD | 21 (28.4%) | 22 (21.8%) |
| Allergic conjunctivitis | 20 (27.0%) | 8 (7.9%) |
| Allergic rhinitis | 32 (43.2%) | 13 (12.9%) |
| Other (atopic dermatitis, urticaria, etc.) | 6 (8.1%) | 11 (10.9%) |
| ≥1 current OCS-related condition | 31 (41.9%) | 46 (45.5%) |
| Hypertension | 19 (25.7%) | 27 (26.7%) |
| Osteoporosis | 6 (8.1%) | 17 (16.8%) |
| Cataract | 5 (6.8%) | 7 (6.9%) |
| Anxiety/Depression | 6 (8.1%) | 5 (5.0%) |
| Type 2 Diabetes Mellitus | 3 (4.0%) | 7 (6.9%) |
| Obstructive sleep apnoea | 3 (4.0%) | 7 (6.9%) |
| Cardiovascular disease | 4 (5.4%) | 3 (3.0%) |
| Other OCS-related ongoing comorbidities | 2 (2.7%) | 17 (16.8%) |
| ≥1 other ongoing comorbidities | 19 (25.7%) | 16 (15.8%) |
| Thyroid disorders | 4 (5.4%) | 4 (4.0%) |
| Bronchiectasis | 3 (4.0%) | 3 (3.0%) |
| **Blood eosinophil count at the index date, cells/mm^3^** | 500 (360-710) | 630 (440-960) |
| **Total serum IgE at the index date, IU/mL (N=61; N=62)** | 390 (150-774.3) | 156 (63-400) |
| **AER (any severity) (N=82; N=114)** | 4.01 | 4.04 |
| **AER for severe exacerbations (N=82; N=114)** | 1.24 | 1 |
| **OCS users** | 19 (22.4%) | 34 (33.7%) |
| **OCS initial dose, prednisone equivalent, mg (N=17; N=31)** | 12.5 (6.3-25) | 10 (5-25) |
| **Lung function at the index date** |  |  |
| Pre-bronchodilator FEV_1_, L (N=58; N=87) | 2.0±0.7 | 1.9±0.8 |
| Post-bronchodilator FEV_1_, L (N=37; N=52) | 2.0±0.8 | 2.2±0.9 |
| Pre-bronchodilator FEV_1_, % predicted (N=61; N=90) | 71.3±22.2 | 70.2±21.9 |
| Post-bronchodilator FEV_1_, % predicted (N=37; N=50) | 72.5±22.8 | 77.4±22.6 |
| Pre-bronchodilator FVC, L (N=56; N=) | 3.1±1.0 | 2.9±1.0 |
| **ACT score at the index date (N=67; N=94)** | 15.4±5.0 | 14.2±4.4 |

*Abbreviations: yrs, years; BMI, body mass index; GERD, gastroesophageal reflux disease; OCS, oral corticosteroids; AER, annual exacerbation rate; FEV_1_, forced expiratory volume in 1 second; FVC, forced vital capacity; ACT, asthma control test.*
