# Supplementary material for: ChAracterization of ItaliaN severe uncontrolled Asthmatic patieNts Key features when receiving Benralizumab in a real-life setting: the observational rEtrospective ANANKE study
Source: Respir Res. 2022 Feb 19;23:36. doi: 10.1186/s12931-022-01952-8 (PMC8858449; doi:10.1186/s12931-022-01952-8)
Supplement: Supplementary file 6 — Additional file 6: Table S2. Evaluable patients with data on OCS use at the index date and at enrolment are 14 for allergic and 30 for non-allergic subjects. OCS dose is indicated as a median (IQR). [file 12931_2022_1952_MOESM6_ESM.docx]

**Additional Table 2.** Evaluable patients with data on OCS use at the index date and at enrolment are 14 for allergic and 30 for non-allergic subjects. OCS dose is indicated as a median (IQR).

|  | Allergic  N=14 | Non-allergic  N=30 |
| --- | --- | --- |
| Dose reduction | 6 (42.9%) | 16 (53.3%) |
| Interruption | 5 (35.7%) | 14 (46.7%) |
| Initial dose, prednisone-equivalent, mg/die | 18.8 (10-0-25.0) | 10 (5.0-25.0) |
| Final dose, prednisone-equivalent, mg/die | 8.1 (0.0-12.5) | 2.3 (0.0-6.0) |
