# Supplementary material for: ChAracterization of ItaliaN severe uncontrolled Asthmatic patieNts Key features when receiving Benralizumab in a real-life setting: the observational rEtrospective ANANKE study
Source: Respir Res. 2022 Feb 19;23:36. doi: 10.1186/s12931-022-01952-8 (PMC8858449; doi:10.1186/s12931-022-01952-8)
Supplement: Supplementary file 7 — Additional file 7: Table S3. Patient characteristics recorded before the start of benralizumab therapy. Data are N (%), mean±SD, or median (IQR). Unless otherwise specified, the evaluable populations included 33 obese, 79 overweight and 70 underweight/normal BMI patients. [file 12931_2022_1952_MOESM7_ESM.docx]

**Additional Table 3.** Patient characteristics recorded before the start of benralizumab therapy. Data are N (%), mean±SD, or median (IQR). Unless otherwise specified, the evaluable populations included 33 obese, 79 overweight and 70 underweight/normal BMI patients.

| **Characteristics** | **Evaluable obese**  **N=33** | **Evaluable overweight**  **N=79** | **Evaluable underweight/normal BMI**  **N=70** |
| --- | --- | --- | --- |
| **Age at the index date, yrs** | 56.5±14.7 | 56.6±12.9 | 55±12.8 |
| **Female sex** | 25 (75.8%) | 38 (48.1%) | 48 (68.6%) |
| **Asthma duration at the index date, yrs (N=32; N=78; N=70)** | 10.2 (7.3-26.6) | 12 (6.3-24.5) | 13.2 (5.4-24.6) |
| **ACT score (N=25; N=61; N=57)** | 14.2±5.1 | 14.6±4.8 | 15.5±4.6 |
| **AER (any severity) (N=32; N=77; N=65)** | 3.72 | 3.99 | 4.49 |
| **AER for severe exacerbations (N=82; N=114; N=70)** | 1.13 | 1.12 | 1.32 |

*Abbreviations: BMI, body mass index; yrs, years; ACT, asthma control test; AER, annual exacerbation rate.*
